# Genetic background influences expression and function of the cation channel TRPM4 in the mouse heart

**DOI:** 10.1007/s00395-020-00831-x

**Published:** 2020-11-17

**Authors:** Rebekka Medert, Andy Pironet, Lucas Bacmeister, Sebastian Segin, Juan E. Camacho Londoño, Rudi Vennekens, Marc Freichel

**Affiliations:** 1 Institute of Pharmacology, Heidelberg University, im Neuenheimer Feld 366, 69120 Heidelberg, Germany; 2 DZHK (German Centre for Cardiovascular Research), Partner Site, Heidelberg/Mannheim, Germany; 3 Laboratory of Ion Channel Research, TRP Research Platform Leuven, VIB Center for Brain and Disease Research, Department of Cellular and Molecular Medicine, KU Leuven, Leuven, Belgium

**Keywords:** Transient receptor potential (TRP) channel, TRPM4, Cardiac contractility, Inotropic response, Catecholamine, Cardiomyocyte specific deletion, Genetic background, C57Bl/6N, 129SvJ

## Abstract

Transient receptor potential melastatin 4 (TRPM4) cation channels act in cardiomyocytes as a negative modulator of the L-type Ca^2+^ current. Ubiquitous Trpm4 deletion in mice leads to an increased β-adrenergic inotropy in healthy mice as well as after myocardial infarction. In this study, we set out to investigate cardiac inotropy in mice with cardiomyocyte-specific Trpm4 deletion. The results guided us to investigate the relevance of TRPM4 for catecholamine-evoked Ca^2+^ signaling in cardiomyocytes and inotropy in vivo in TRPM4-deficient mouse models of different genetic background. Cardiac hemodynamics were investigated using pressure–volume analysis. Surprisingly, an increased β-adrenergic inotropy was observed in global TRPM4-deficient mice on a 129SvJ genetic background, but the inotropic response was unaltered in mice with global and cardiomyocyte-specific TRPM4 deletion on the C57Bl/6N background. We found that the expression of TRPM4 proteins is about 78 ± 10% higher in wild-type mice on the 129SvJ versus C57Bl/6N background. In accordance with contractility measurements, our analysis of the intracellular Ca^2+^ transients revealed an increase in ISO-evoked Ca^2+^ rise in Trpm4-deficient cardiomyocytes of the 129SvJ strain, but not of the C57Bl/6N strain. No significant differences were observed between the two mouse strains in the expression of other regulators of cardiomyocyte Ca^2+^ homeostasis. We conclude that the relevance of TRPM4 for cardiac contractility depends on homeostatic TRPM4 expression levels or the genetic endowment in different mouse strains as well as on the health/disease status. Therefore, the concept of inhibiting TRPM4 channels to improve cardiac contractility needs to be carefully explored in specific strains and species and prospectively in different genetically diverse populations of patients.

## Introduction

During cardiac contraction cardiomyocytes of the working myocardium undergo electro-mechanical coupling. With extracellular electrical excitation, voltage-gated L-Type Ca^2+^ channels in the membrane of the transverse tubules opens followed by a Ca^2+^ influx, which elevates [Ca^2+^]_i_ and thereby activates calcium-induced calcium release (CICR) initiating myocardial contraction [4, 8]. Cardiac contractility is influenced by numerous regulatory mechanisms, including the adjustment by the sympathetic nervous system. β-adrenergic stimulation by catecholamines activates the adenylyl cyclase (AC) which converts ATP to cAMP. An elevation in cytosolic cAMP level in cardiomyocytes leads to the activation of cAMP-dependent protein kinase A (PKA), which increases the activity of various effector proteins [20, 32]. PKA phosphorylation of the voltage-gated L-type Ca^2+^ channel, the ryanodine receptor RyR2 and Phospholamban, which relieves the inhibition of the sarcoplasmic reticulum (SR) Ca^2+^ ATPase SERCA2a, increases the intracellular Ca^2+^ influx during systole and provide more rapid reuptake of Ca^2+^ ions into the sarcoplasmic reticulum during diastole [4, 15]. Consequently, in the following systole, a larger Ca^2+^ transient is generated resulting in a higher contraction force [29].

Expression of the calcium-activated monovalent cation channel Transient Receptor Potential Melastatin 4 (TRPM4) has been found in the heart of humans as well as in the atrium and ventricle of the mouse heart. It is expressed in cardiomyocytes, cardiac conduction system as well as in endothelial cells and smooth muscle cells [5, 11, 16, 17, 22, 26]. The gain of function mutations of the Trpm4 gene are strongly associated with familial arrhythmia syndromes such as Brugada syndrome and the progressive familial heart block type I [16, 18, 34]. Insights from Trpm4-deficient cardiomyocytes have identified TRPM4 as a negative modulator of the L-type Ca^2+^ influx under β-adrenergic stimulation, and its deletion leads to an increased action potential duration and thereby results in an enhanced driving force for Ca^2+^ through the L-Type calcium channel. Consequently, systolic Ca^2+^ transients are elevated under β-adrenergic stimulation, and contractility of isolated left ventricular papillary muscles showed that β-adrenergic stimulation leads to an increased contractile force in TRPM4-deficient mice compared to wildtype controls [21, 37]. Furthermore, hemodynamic measurements under increasing β-adrenergic stimulation with isoproterenol resulted in a stronger left ventricular inotropic response in TRPM4-deficient mice compared to controls. However, under basal conditions, no significant differences were reported [21]. In addition, an enhanced β-adrenergic cardiac reserve was observed in TRPM4-deficient mice under conditions of ischemia evoked heart failure compared to wildtype controls [10]. These studies suggest a pharmacological or genetic inactivation of TRPM4 as a potential treatment strategy to enhance cardiac output in patients with severe heart failure with elevated catecholamine levels.

## Methods

### Mice and genotyping

Experimental procedures were approved by the regional council Karlsruhe according to the Animal Welfare Act (T-64/18, G-89/15), and by the Animal Ethics Committee of the KU Leuven (In Vitro-Vennekens). TRPM4-deficient mouse lines were described previously [39]. Breeding and housing of TRPM4-deficient mice and littermate controls were performed in the Interfaculty Biomedical Faculty (IBF) of the University of Heidelberg. Mice were kept under specified pathogen-free conditions on a 12-h light/12-h dark cycle with water and standard food (Rod18, LASvendi GmbH, Germany) available ad libitum. Experiments were performed in 18–19 weeks old male mice. Trpm4^+/–^ mice [39] were independently backcrossed to the 129SvJ and C57Bl/6N background for 6 and 10 generations respectively, before Trpm4^+/–^ mice on either background were bred to obtain Trpm4^–/–^ mice and corresponding Trpm4^+/+^ litter matched controls (termed Trpm4^–/–(Bl/6N)^ and Trpm4^+/+(Bl/6N)^ throughout the manuscript). Trpm4^–/–(129SvJ)^ and Trpm4^+/+(129SvJ)^ littermatched control mice on the 129SvJ background were obtained by analogous breeding. The generation of Trpm4^+/flox^ mice was also reported previously [39]. After 9 generations of backcrossing to the C57Bl/6N strain, Trpm4^+/flox^ mice were mated with αMHC-Cre^ERT2^ mice [36] (also on the C57Bl/6N background). To induce Cre recombinase-mediated deletion of exons 15 and 16 of the Trpm4 gene selectively in cardiomyocytes of adult mice, a daily tamoxifen injection was performed on five consecutive days on 6 weeks old Trpm4^flox/flox^; αMHC-CreERT2 positive (Cre^pos^) mice (termed Trpm4^iCM−KO^). Trpm4^flox/flox^; αMHC-CreERT2 negative (Cre^neg^) littermates were treated with tamoxifen as well and served as controls.

The identification of the genotype of the experimental mice was carried out by PCR using the primer pairs listed in Table 1. Ear biopsies were transferred into 97.5 μl of DirectPCR buffer (Viagen, USA) with 2.5 μl proteinase K (10 mg/ml, AppliChem, Germany) and lysed overnight at 55 °C. Subsequently, the proteinase K was denatured for 45 min at 85 °C.

**Table 1:** Primer sets for genotyping

Allele	Primer sequence
Trpm4 WT	5´ GTTTGATGTCTCCTTCAGTCG 3´ 5´ ACCTACAGGAAACCTCGGGG 3´
Trpm4 ^–^	5´ GTTTGATGTCTCCTTCAGTCG 3´ 5´ GAGTTCCTGTCCTCCTAAAGG 3´
Trpm4^flox^	5´ GTTTGATGTCTCCTTCAGTCG 3´ 5´ ACCTACAGGAAACCTCGGGG 3´
αMHC-Cre^ERT2^	5´ TTATGGTACCACATAGACCTCT 3´ 5´ TGCTGTTGGATGGTCTTCACAG 3´

### Microsomal membrane preparation

Mice were sacrificed using cervical dislocation. The thorax was opened and the still-beating heart was washed out with NaCl 0.9% (Braun, Germany) injected via the apex. Mouse hearts were transferred in PBS and atria were removed. Ventricular tissue was homogenized in 1 ml lysis buffer (100 mM TRIS–HCl; 1 mM MgCl_2_; pH 8.0 supplemented with 1 mM Iodacetamide; 1 mM Phenantholin; 0.1 mM PMSF; 1 µg/ml Antipain; 1 µg/ml Leupeptin; 0.7 µg/ml Pepstatin; 1 mM Benzamidine; 0.3 µM Aprotinin, Applichem, Germany) using a 2 ml tissue grinder (Tenbroeck Tissue Grinders, Wheaton). Homogenates were filled up to 3 ml with lysis buffer and were frozen at − 80 °C for 20 min following by thawing on ice for about 1 h. Afterwards 15 ml of sucrose buffer (0.25 M sucrose; 10 mM TRIS–HCl; pH 7.4 supplemented with 1 mM Iodacetamide; 1 mM Phenantholin; 0.1 mM PMSF; 1 µg/ml Antipain; 1 µg/ml Leupeptin; 0.7 µg/ml Pepstatin; 1 mM Benzamidine; 0.3 µM Aprotinin, Applichem, Germany) was added and the samples were centrifuged for 30 min at 6000×*g*. Subsequently, supernatant was centrifuged at 50.000×*g* for 1 h 45 min. The resulting microsomal membrane pellet was solved in 200 µl sucrose buffer. Protein concentration was determined by BCA assay (Thermofisher Scientific USA; 23227). Microsomal membrane fractions were supplemented with 4 × Laemmli buffer (60 mM TRIS–HCl; 10% (v/v) Glycerol; 5% (v/v) β-mercaptoethanol; 4% (w/v) SDS; 0.005% (w/v) Bromophenol blue; pH 6.8) and incubated at 60 °C for 20 min. Afterwards microsomal membrane fractions were stored at − 80 °C.

### Immunoblotting

Microsomal membrane fractions (50 µg/well) were loaded on a 10% Bis–Tris Plus gel (Invitrogen, USA; NW04122) followed by gel electrophoresis at 130 V for 90 min. Resolved proteins were electroblotted onto a 0.45 µm Protran nitrocellulose membrane (GE Healthcare, USA; A10195922) at 12 mV for 60 min. Afterwards the membrane was blocked in 5% non-fat milk with TBST (50 mM TRIS–HCl; 150 mM NaCl; 0.1% (v/v) Tween-20; pH 7.5). Blots were incubated in anti-GAPDH 1:500 (Acris, USA; ACR001P), anti-TRPM4 [39] 1:100 (ab578 provided by Veit Flockerzi), anti-Ca_v_β_2_ [23] 1:100 (ab425 provided by Veit Flockerzi) or anti-SERCA2a (Badrilla, UK; A010-23L) at 4 °C for 24 h. Blots were washed three times for 10 min in TBST and subsequently incubated in horseradish peroxidase-conjugated anti-rabbit 1:50.000 (GE Healthcare, USA; NA9340V) at RT for 2 h. Blots were washed two times for 10 min in TBST and once for 10 min in TBS (50 mM TRIS–HCl; 150 mM NaCl; pH 7.5). Blots were incubated in SignalFire Elite ECL (Cell Signaling, USA) for 1 min and chemoluminescence was detected by digital imaging (GE Healthcare, ImageQuant LAS 4000 mini). Protein expression levels were determined by densitometry analysis using ImageJ software.

### Cardiac hemodynamic measurements

Mouse ventricular function was measured by a miniaturized pressure-conductance catheter. Mice were anesthetized by peritoneal injection of an anesthetic-analgesic mixture of 1200 mg/kg Urethane (Sigma-Aldrich, USA), 50 mg/kg α-Chloralose (Sigma-Aldrich, USA), and 0.1 mg/kg Buprenorphine (Bayer AG, Germany). Anesthetized mice were intubated and mechanically ventilated with 100% O_2_ by a small rodent respirator (MiniVent 845, Harvard Apparatus, USA). Body temperature was monitored using a rectal temperature probe (Hugo Sachs Electronic, Germany) and maintained at 37 ± 0.2 °C by placing the mouse on a heating plate (Hotplate 062, Labotect). Upon the absence of interdigital reflexes, 1 μg/g of the muscle relaxant Pancuronium (Sigma-Aldrich, USA) was administered intraperitoneally. A central venous catheter was placed in the left femoral vein for continuous infusion (15 μl/min; 11Plus, Harvard Apparatus, USA) of NaCl 0.9% (Braun, Germany) and for the application of different isoproterenol concentrations for β-adrenergic stimulation during the measurement [1]. After performing a limited thoracotomy with an electric cautery pen (Faromed, Germany), the Pressure–Volume loop (PV loop) catheter SPR-839 (Millar, USA) was placed into the mouse left ventricular cavity via the apex. Optimal placement of the catheter was followed by a stabilization phase of 10 min. Preload dependent parameters were recorded by an expiratory ventilation break of max. 5 s. To assess preload independent contractility, the inferior caval vein was occluded and five consecutive PV loops were recorded during preload reduction to analyze Preload-Recruitable Stroke Work (PRSW). After basal recordings followed the β-adrenergic stimulation by infusion of increasing concentrations of the positive inotropic substance isoproterenol (9, 30, (60), 90 to a maximum of 300 ng/kg/min) [21, 27]. At the end of each measurement, the parallel conductance of the myocardium was determined by an intravenous bolus injection of 15 μl of a 15% hypertonic NaCl solution. Cuvette calibration of heparinized blood from each mouse was used for conductance (µS) to blood volume (µl) conversion.

### Isolation of ventricular cardiac myocytes

10- to 12 weeks-old mice were heparinized and sacrificed by intraperitoneal injection of sodium pentobarbital (Dolethal, Vétoquinol, UK). Hearts were quickly removed and perfused retrograde through the aorta with O_2_-saturated Ca^2+^-free Tyrode solution containing (in mM): 117 NaCl, 4 KCl, 1 KH_2_PO_4_, 4 NaHCO_3_, 10 HEPES, 10 Glucose and 1.7 MgCl_2_ (pH = 7.4 with NaOH). Afterwards, the heart was washed with Tyrode solution containing 0.23 mM EGTA. This step was followed by 5–10 min perfusion of Tyrode solution containing 100 µM Ca^2+^ and Collagenase B (Worthington). Next, the heart was removed from the Langendorff apparatus, atria were discarded and ventricular muscle was cut into small pieces in fresh enzyme solution supplemented with 300 µM Ca^2+^. This suspension was gently agitated for 2–3 min, filtered through a 100 µM nylon mesh and centrifuged at 400 rpm for 3 min. The resulting pellet was washed with Tyrode solution containing 0.5 mM Ca^2+^ and BSA. The suspension was left for decantation for 3–7 min and the pellet was washed again with Tyrode solution containing 1 mM Ca^2+^ and BSA. Myocytes were kept at room temperature and used for experiments for 7 h**.**

### Calcium imaging

Cardiomyocytes were loaded with 5 µM Fluo-4 AM for 30 min at 37 °C. After placing Fluo4- loaded cells in a recording chamber, cells were constantly perfused with an extracellular solution containing (in mM): 95 NaCl, 4.8 KCl, 24.88 NaHCO_3_, 1.18 KH_2_PO_4_, 10 Glucose, 1.5 CaCl_2_ and 1.87 MgCl_2_. This solution was continuously bubbled with carbogen (5% CO_2_, 95% O_2_). Measurements were performed at a temperature of 32 °C using a single channel bipolar temperature controller (CL-100) (Warner Instruments, USA). Electrical stimulation was achieved via local field stimulation using a custom-made electrode. Stimulation was provided by 2 ms pulses of 3 V at 5 Hz using a MyoPacer EP Field stimulator (IonOptix, Netherlands). Fluorescence was detected using a photo-multiplier tube and was acquired by the Patchmaster (v2 × 75) software (HEKA Elektronik, Germany). Excitation light (477 nm) was provided by a Polychrome V monochromator (TILL Photonics, Germany). Data were sampled at 5 kHz. Fluorescence is shown as: F = (F–F_1)/F_0, with F0 = basal Ca^2+^-signal, and F1 = Ca^2+^-signal 2 ms before the occurrence of each Ca^2+^-transient. In the analysis, F2 represented the peak fluorescence of the Ca^2+^-transient. The results are displayed as averaged values from cells of biologically independent mice (10 WT and 9 KO animals on C57Bl/6N and 8 WT and 10 KO animals on 129SvJ background, respectively).

### Statistics

Statistical analyses were performed using OriginPro 8.5 and Excel 2010. Data are expressed as mean ± standard error of the mean (SEM) or mean ± standard deviation (SD). Normality distribution of the data was tested using a Shapiro–Wilk test and considered normally distributed if *p* > 0.05. The difference between the two groups was tested using a Two-Sample *T*-test or its non-parametric alternative, Mann–Whitney test. *P*-values < 0.05 were considered statistically significant.

## Results

**Fig. 1:**
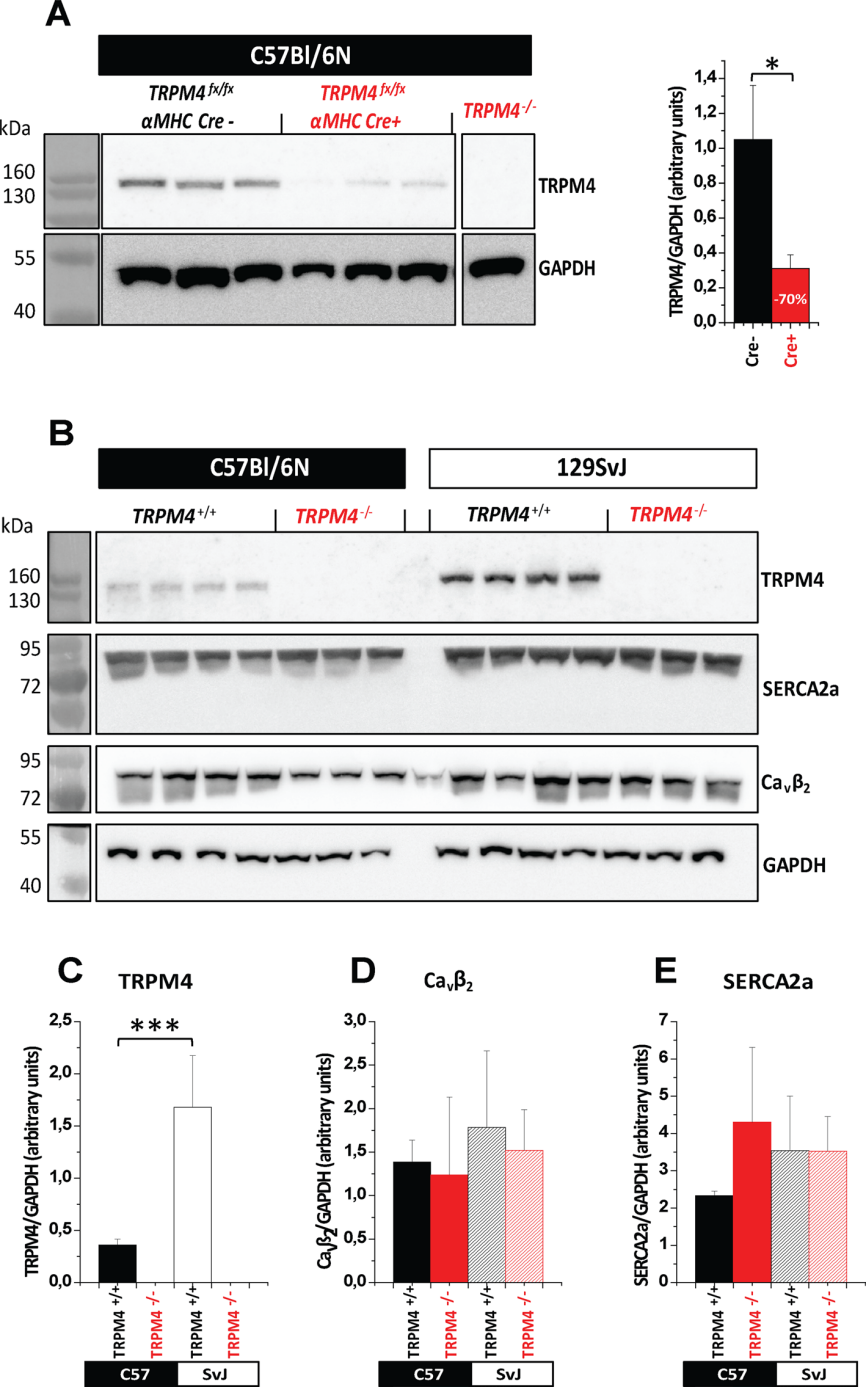
Protein expression analysis of TRPM4 in adult Trpm4^flox/flox^; αMHC-Cre^ERT2^ mice and constitutive Trpm4-deficient mice on the genetic C57Bl/6N or 129SvJ background. **a** Western blots (left) with anti-TRPM4 and anti-GAPDH, 10 weeks after tamoxifen treatment; right panel: Analysis of the relative expression analysis shows a TRPM4 reduction of 78 ± 10% in the heart of Trpm4^flox/flox^; αMHC-CreERT2 ^pos.^ animals. Microsomal membrane fractions from *n* = 4 independent Cre negative and Cre positive mice were analyzed. **b** Western blot analysis with anti-TRPM4, anti-SERCA2a, anti-Ca_v_β_2_ and anti-GAPDH in globally Trpm4^–/–(Bl/6N)^ (*n* = 4) and Trpm4^–/–(129SvJ)^ (*n* = 3) mice compared to Trpm4^+/+(Bl/6N)^ (*n* = 4) and Trpm4^+/+(SvJ)^ (*n* = 3) littermate controls. Relative expression of TRPM4 (**c**), Ca_v_β_2_ (**d**) and SERCA2a (**e**). Trpm4^+/+(Bl/6N)^ Values are expressed as means ± SD. *p < 0.05, **p < 0.01

**Fig. 2:**
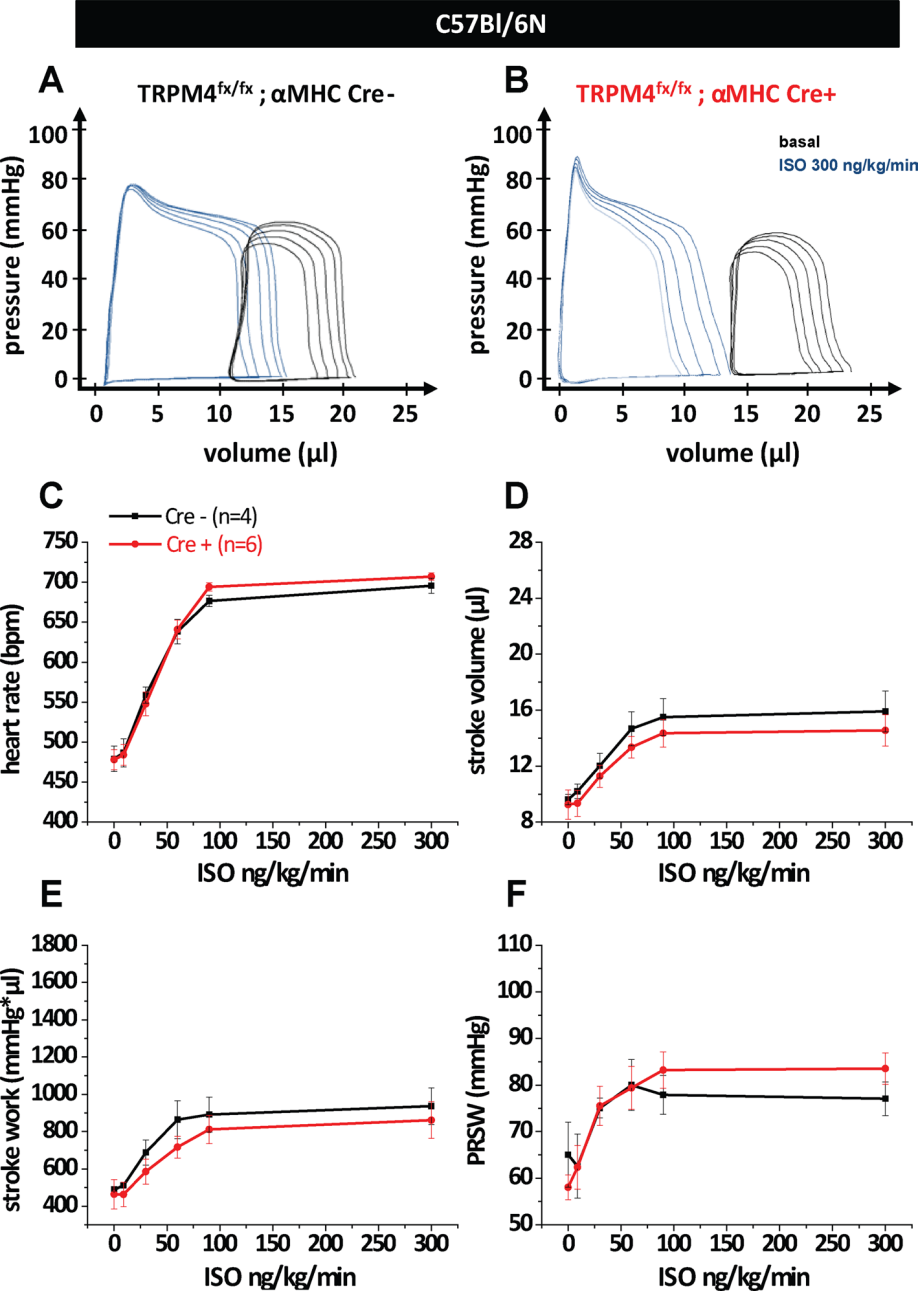
Measurement of cardiac function of inducible and cardiomyocyte-specific TRPM4-deficient mice on the C57Bl/6N genetic background. Representative pressure–Volume loops under basal conditions and β-adrenergic stimulation of **a** Trpm4^flox/flox^/αMHC-Cre^ERT2^ negative (*n* = 4) and **b** Trpm4^flox/flox^/αMHC-Cre^ERT2^ positive (*n* = 6) littermates. Statistical analysis of isoproterenol dose–response curves of **c** heart rate, **d** stroke volume, **e** stroke work and **f** PRSW (Preload-Recruitable-Stroke Work). Values are expressed as means ± SEM

Previous data from our group indicate that TRPM4 global knockout mice have an improved cardiac inotropic response to β-adrenergic stimulation [21]. To test whether the deletion of the Trpm4 gene specifically in cardiomyocytes improves cardiac contractility in healthy mice, we generated mice with a αMHC-Cre dependent deletion of the Trpm4 gene. To analyze the efficiency of cardiomyocyte-specific Trpm4 deletion in Trpm4^flox/flox^; αMHC-Cre^pos^ (Trpm4^iCM−KO^) mice, we isolated microsomal membrane fractions from hearts of three independent Trpm4^flox/flox^; αMHC-Cre^pos^ and Trpm4^flox/flox^; αMHC-Cre^neg^ mice, respectively, and performed Western Blot analysis. Proteins of ∼ 136 kDa were detected in the heart of Trpm4^flox/flox^; αMHC-Cre^neg^ using ab478, which identified TRPM4 specifically in other cells and tissues from WT but not from the corresponding Trpm4^–/–^ controls [22]. The size of these proteins is within the expected size range for TRPM4 proteins and the expression level of these proteins was reduced by 78 ± 10% in Trpm4^iCM−KO^ mice. Trpm4^–/–^ mice entirely lacking TRPM4 proteins served as control (Fig. 1a). Using these mice, cardiac function was assessed using Pressure–Volume loop (PV-loop) measurements. After recordings of basal heart function, acute β-adrenergic stimulation with isoproterenol in Trpm4^iCM−KO^ and Cre^neg^ control mice was performed (Fig. 2a and b). A gradual increase in isoproterenol concentrations from 9 to 300 ng/kg/min resulted in the expected rise in heart rate (Fig. 2c) and systolic parameters such as stroke volume (Fig. 2d) stroke work (Fig. 2e) and the contractility parameter Pre-recruitable Stroke Work (PRSW) (Fig. 2f). Additional parameters describing cardiac function were analyzed and are listed in Table 2. However, the results of the functional measurements showed no significant difference in cardiac contractile function between Cre^pos^ and Cre^neg^ animals.

**Table 2:** Hemodynamic analysis of inducible cardiomyocyte-specific Trpm4^flox/flox^; αMHC-Cre^ERT2^ mice. Cre^neg^, *n* = 4; Cre^pos^, *n* = 6

	C57Bl/6N											
Isoproterenol (ng/kg/min)	0		9		30		60		90		300	
Genotype: Trpm4^flox/flox^	Cre-	Cre +	Cre-	Cre +	Cre-	Cre +	Cre-	Cre +	Cre-	Cre +	Cre-	Cre +
HR (b/min)	479 ± 16	478 ± 12	487 ± 18	484 ± 13	559 ± 10	548 ± 15	638 ± 15	641 ± 12	677 ± 17	694 ± 5	696 ± 9	707 ± 5
SV (µl)	9.6 ± 0.4	9.3 ± 1.0	10.2 ± 0.5	9.4 ± 1.0	12.0 ± 0.9	11.3 ± 0.8	14.7 ± 1.2	13.3 ± 0.8	15.5 ± 1.7	14.3 ± 1.0	15.9 ± 1.5	14.6 ± 1.1
CO (µl/min)	4604 ± 214	4392 ± 450	4967 ± 312	4515 ± 445	6704 ± 451	6190 ± 491	9325 ± 641	8529 ± 406	10,460 ± 1071	9943 ± 635	11,075 ± 1083	10,298 ± 822
Ved (µl)	26.6 ± 3.5	23.2 ± 2.5	25.9 ± 3.2	22.9 ± 2.4	22.0 ± 2.7	18.6 ± 2.0	17.9 ± 2.1	15.1 ± 1.7	16.9 ± 2.1	14.0 ± 1.8	16.8 ± 2.0	14.5 ± 1.8
Ves (µl)	18.3 ± 3.7	15 ± 9	17.6 ± 3.3	15.5 ± 2.4	11.7 ± 2.3	9.5 ± 2.3	4.0 ± 1.2	2.6 ± 1.7	2.2 ± 1.0	1.4 ± 1.0	2.6 ± 1.3	1.6 ± 1.2
Ped (mmHg)	3.7 ± 1.0	2.7 ± 0.8	3.5 ± 0.8	2.8 ± 0.7	4.3 ± 2.1	2.1 ± 0.7	2.8 ± 0.7	1.9 ± 0.8	2.8 ± 0.6	1.4 ± 1.5	4.2 ± 1.9	2.5 ± 1.1
Systolic parameters												
SW (mmHg*s)	489 ± 7	464 ± 79	511 ± 12	463 ± 66	689 ± 68	585 ± 67	864 ± 102	717 ± 59	892 ± 93	812 ± 75	937 ± 99	862 ± 98
dP/dt_max_ (mmHg/s)	393 ± 229	3711 ± 196	4113 ± 316	3800 ± 160	5648 ± 361	4862 ± 394	8205 ± 964	6768 ± 234	9733 ± 465	9015 ± 280	11,763 ± 1303	10,241 ± 449
PRSW (mmHg)	65.0 ± 0.9	58.0 ± 0.9	62.6 ± 1.4	62.3 ± 1.2	75.1 ± 1.0	75.5 ± 1.0	8.0 ± 0.8	79.4 ± 1.0	78.9 ± 0.7	83.2 ± 0.5	77.1 ± 0.6	83.5 ± 0.2
Diastolic parameters												
-dP/dt_min_ (mmHg/s)	– 4827 ± 417	– 4467 ± 211	– 5012 ± 571	– 4686 ± 228	– 6657 ± 1048	– 5492 ± 365	– 6643 ± 587	– 5994 ± 541	– 5969 ± 532	– 6289 ± 569	– 7793 ± 1491	– 6488 ± 526
Tau (ms)	8.3 ± 0.6	8.0 ± 0.4	7.7 ± 0.4	7.7 ± 0.4	5.7 ± 0.3	5.8 ± 0.3	4.9 ± 0.6	4.6 ± 0.2	4.7 ± 0.5	4.5 ± 0.3	4.6 ± 0.4	4.6 ± 0.2

HR, heart rate; SV, stroke volume; CO, cardiac output; Ved, end-diastolic volume; Ves, end-systolic volume; Ped, end-diastolic pressure; SW, stroke work; dP/dt_max_, peak rate of pressure rise; -dP/dt_min_, peak rate of pressure decline; PRSW, preload recruited stroke work; Tau, relaxation time constant (Weiss method). Values are expressed as means ± SEM

**Fig. 3:**
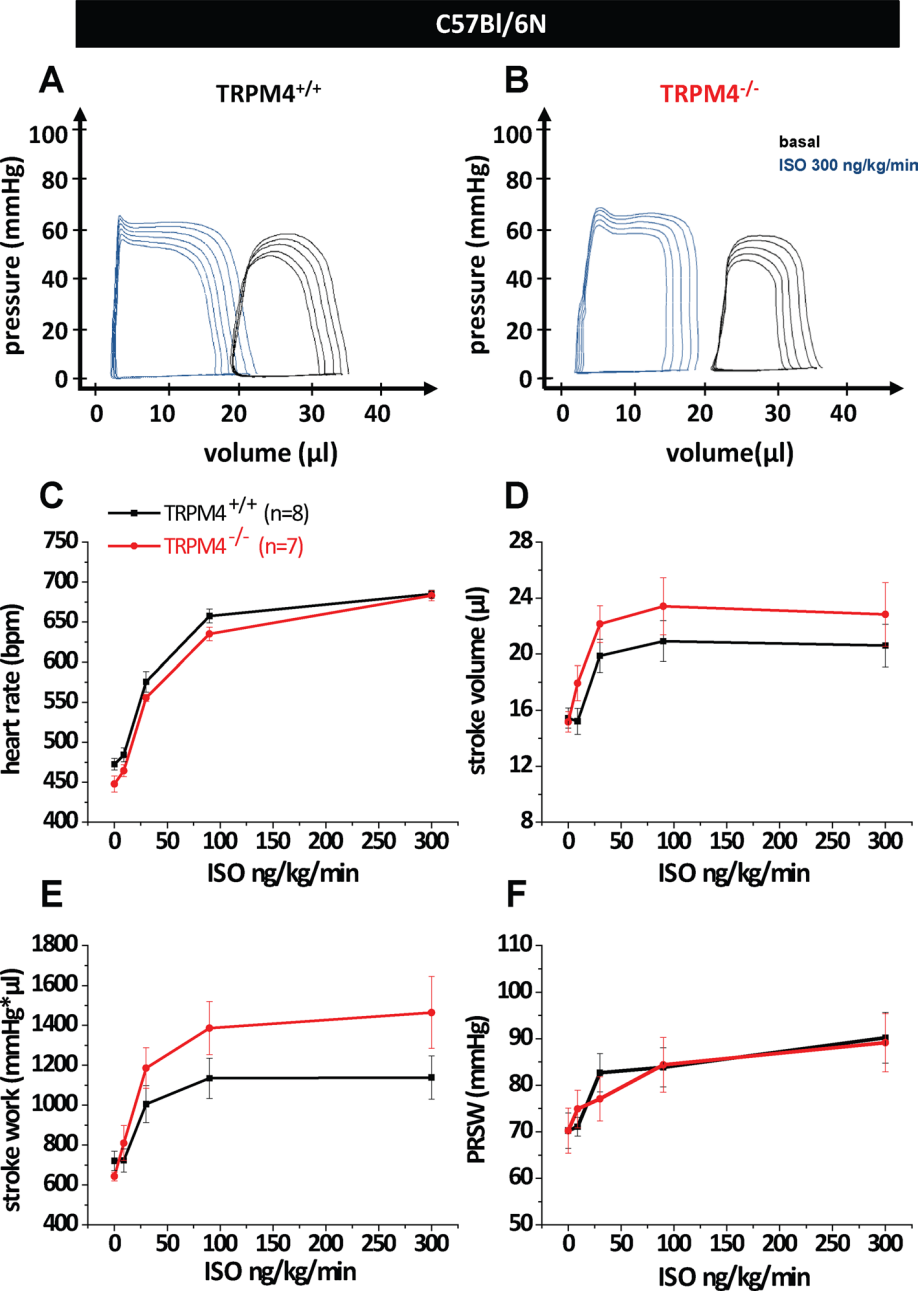
Measurement of cardiac function in global TRPM4 knockout mice on the C57Bl/6N genetic background. Representative pressure–Volume loops under basal conditions and β-adrenergic stimulation of **a** Trpm4^+/+(Bl/6N)^ mice (*n* = 8) and **b** Trpm4^–/– (Bl/6N)^ (*n* = 7) littermates. Statistical analysis of isoproterenol dose–response curves of **c** heart rate, **d** stroke volume, **e** stroke work and **f** PRSW (Preload-Recruitable-Stroke Work). Values are expressed as means ± SEM

Since we did not find a difference in mice lacking Trpm4 selectively in cardiomyocytes we analyzed Trpm4 global knockout mice on the same C57Bl/6N genetic background (Trpm4^–/–(Bl/6N)^ and litter matched Trpm4^+/+(Bl/6N)^ as controls) as we did with the Trpm4^iCM−KO^ before. Western Blot analysis of microsomal membrane fractions of Trpm4^+/+(Bl/6N)^ again showed a single band (∼ 136 kDa) whereas these proteins were not detected in the corresponding Trpm4^–/–(Bl/6N)^ hearts (Fig. 1a, c). After measuring the basal cardiac function, acute β-adrenergic stimulation via the venous catheter followed. A gradual increase in isoproterenol concentrations from 9 to 300 ng/kg/min led to the expected β-adrenergic dose–response relationship in TRPM4^−/−(Bl/6N)^ mice as well as in TRPM4^+/+(Bl/6N)^ littermates (Fig. 3a and f). Again, the analysis of the functional measurements showed no significant differences in heart rate (Fig. 3c) nor in the systolic parameters stroke volume (Fig. 3d), stroke work (Fig. 3e) or the contractility parameter PRSW (Fig. 3f) between TRPM4^−/−(Bl/6N)^ and TRPM4^+/+(Bl/6N)^ mice. Additional parameters describing cardiac function were analyzed and are listed in Table 3.

**Table 3:** Hemodynamic analysis of constitutively TRPM4-deficient mice on the genetic C57Bl/6 N background. TRPM4^+/+^, *n* = 8; TRPM4^−/−^, *n* = 7

	C57Bl/6N									
Isoproterenol (ng/kg/min)	0		9		30		90		300	
Genotype: Trpm4	+ / +	-/-	+ / +	-/-	+ / +	-/-	+ / +	-/-	+ / +	-/-
HR (b/min)	473 ± 7	448 ± 10	484 ± 8	464 ± 7.5	575 ± 12	556 ± 4	657 ± 8	635 ± 8	685 ± 4	683 ± 6.4
SV (µl)	15.5 ± 0.7	15.2 ± 0.7	14.9 ± 0.9	18.0 ± 1.2	19.9 ± 1.1	22.1 ± 1.3	20.9 ± 1.5	23.4 ± 2.1	20.6 ± 1.5	22.8 ± 2.3
CO (µl/min)	7305 ± 377	6786 ± 29	7377 ± 495	8332 ± 618	11,423 ± 699	12,295 ± 102	13,716 ± 892	14,897 ± 1398	14,108 ± 1052	15,639 ± 1664
Ved (µl)	33.7 ± 1.6	37.7 ± 2.4	33.1 ± 2.0	37.2 ± 2.0	25.2 ± 2.2	28.4 ± 3.0	21.5 ± 1.8	24.0 ± 2.3	21.6 ± 1.6	23.5 ± 2.0
Ves (µl)	21.8 ± 1.4	26.8 ± 2.1	21.0 ± 1.9	24.0 ± 1.3	6.7 ± 2.1	8.0 ± 1.7	1.5 ± 0.5	1.7 ± 0.7	3.9 ± 0.6	3.1 ± 0.9
Ped (mmHg)	3.6 ± 0.5	4.8 ± 0.3	3.7 ± 0.5	5.2 ± 0.5	3.2 ± 0.4	4.5 ± 0.5	3.9 ± 0.7	4.4 ± 0.7	5.3 ± 1.4	5.0 ± 0.7
Systolic parameters										
SW (mmHg*s)	721 ± 48	643 ± 29	723 ± 57	810 ± 88	1005 ± 93	1186 ± 102	1135 ± 101	1386 ± 133	1139 ± 109	1464 ± 180
dP/dt_max_ (mmHg/s)	3580 ± 169	2897 ± 104	3654 ± 206	3547 ± 321	5853 ± 573	5471 ± 344	8803 ± 481	7913 ± 485	9698 ± 467	9668 ± 438
PRSW (mmHg)	70.2 ± 3.7	70.2 ± 4.9	71.1 ± 2.0	75.0 ± 4.0	82.7 ± 4.1	77.1 ± 4.7	83.8 ± 4.2	84.4 ± 5.9	90.2 ± 5.5	89.1 ± 6.2
Diastolic parameters										
dP/dt_min_ (mmHg)	-4061 ± 224	-3182 ± 153	-4082 ± 223	-3854 ± 302	-4907 ± 251	-5308 ± 220	-5690 ± 270	-5708 ± 375	-6766 ± 418	-6445 ± 478
Tau (ms)	9.0 ± 0.3	10.0 ± 0.4	8.7 ± 0.4	8.7 ± 0.5	5.8 ± 0.2	5.8 ± 0.2	5.4 ± 0.3	1.5 ± 0.7	4.9 ± 0.1	5.2 ± 0.3

HR, heart rate; SV, stroke volume; CO, cardiac output; Ved, end-diastolic volume; Ves, end-systolic volume; Ped, end-diastolic pressure; SW, stroke work; dP/dt_max_, peak rate of pressure rise; -dP/dt_min_, peak rate of pressure decline; PRSW, preload recruited stroke work; Tau, relaxation time constant (Weiss method). Values are expressed as means ± SEM

**Fig. 4:**
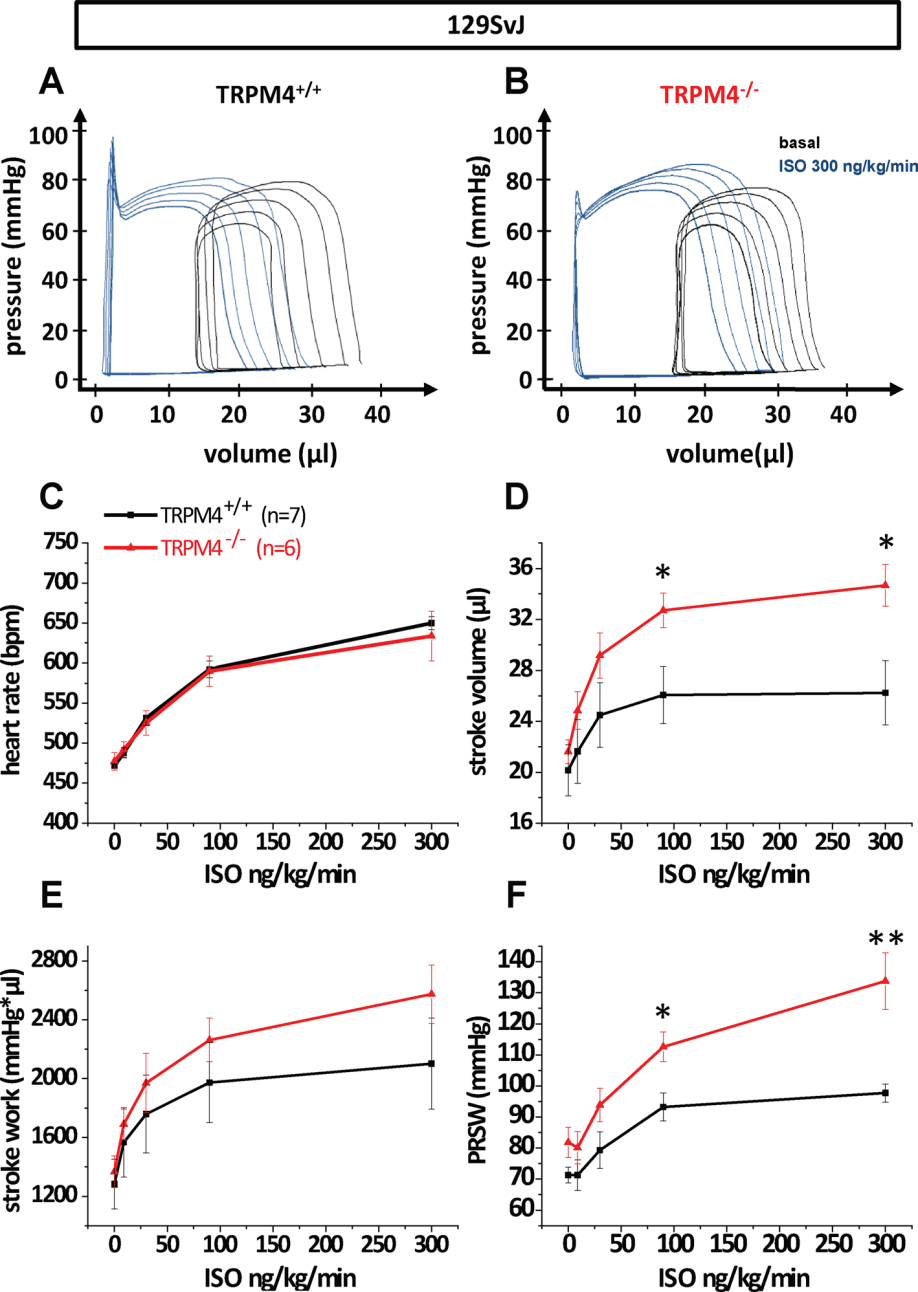
Measurement of cardiac function in global TRPM4 knockout mice on the 129SvJ genetic background. Representative pressure–Volume loops under basal conditions and β-adrenergic stimulation of **a** Trpm4^+/+(129SvJ)^ mice (*n* = 7) and **b** Trpm4^–/–(129SvJ)^ (*n* = 6) littermates. Statistical analysis of isoproterenol dose–response curves of **c** heart rate, **d** stroke volume, **e** stroke work and **f** PRSW (Preload-Recruitable-Stroke Work). Values are expressed as means ± SEM. *p < 0.05, **p < 0.01

Since all cardiac functional parameters analyzed were unaltered in Trpm4^–/–(Bl/6N)^ mice compared to corresponding Trpm4^+/+(Bl/6N)^ controls, we wanted to determine whether the differences to previously published results showing increased contractility in Trpm4^–/–^ mice on a 129SvJ background upon isoproterenol treatment in PV loop measurements [21] and isolated papillary muscle preparations [37], were due to the different genetic background. Thus, we evaluated Trpm4^–/–(129SvJ)^ mice in comparison to litter matched Trpm4^+/+(129SvJ)^ controls. Western Blot analysis in this mouse line showed an abundant expression of ∼ 136 kDa proteins in Trpm4^+/+(129SvJ)^ mice, whereas these proteins were absent in Trpm4^–/–(129SvJ)^ mice (Fig. 1b, c). Again, measurements of basal heart function were followed by acute β-adrenergic stimulation via infusion of increasing isoproterenol concentrations from 9 to 300 ng/kg/min, which resulted in expected dose-dependent changes in PV loops in TRPM4^−/−(129SvJ)^ mice as well as in TRPM4^+/+(129SvJ)^ littermates (Fig. 4a and f). The evaluation of the functional parameters showed no change in heart rate between TRPM4^−/−(129SvJ)^ and TRPM4^+/+(129SvJ)^ littermates (Fig. 4c). In contrast, a significantly increased β-adrenergic inotropic response in stroke volume (Fig. 4d) and in the left ventricular contractility parameter PRSW (Fig. 4f), which represents a key parameter to characterize cardiac function independent of preload and afterload [1, 9], was found in TRPM4^−/−(129SvJ)^ mice compared to TRPM4^+/+(129SvJ)^ control mice. Additional parameters describing cardiac function were analyzed and are listed in Table 4.

**Table 4:** Hemodynamic analysis of constitutively TRPM4-deficient mice on the genetic 129SvJ background. TRPM4^+/+^, *n* = 7; TRPM4^−/−^, *n* = 6

	129SvJ									
Isoproterenol (ng/kg/min)	0		9		30		90		300	
Genotype: Trpm4	+ / +	-/-	+ / +	-/-	+ / +	-/-	+ / +	-/-	+ / +	-/-
HR (b/min)	471.9 ± 2	477 ± 11	488 ± 6	492 ± 10	531 ± 9	526 ± 15	592 ± 10	590 ± 19	650 ± 8.3	634 ± 31
SV (µl)	20.2 ± 2.0	21.6 ± 0.9	21.6 ± 2.5	24.9 ± 1.5	24.5 ± 2.5	29.2 ± 1.8	26.1 ± 2.3	*32.7 ± 1.4	26.2 ± 2.5	*34.6 ± 1.7
CO (µl/min)	9500 ± 931.3	10,338 ± 610	11,500 ± 1505	12,249 ± 824	14,310 ± 1895	15,408 ± 1246	17,111 ± 272	19,257 ± 888	19,068 ± 309	21,784 ± 530
Ved (µl)	37.0 ± 4.1	40.4 ± 1.7	36.4 ± 4.6	42.8 ± 2.3	35.1 ± 3.7	39.4 ± 2.5	30.8 ± 3.8	34.8 ± 2.2	30.8 ± 4.0	35.4 ± 1.8
Ves (µl)	19.1 ± 2.5	23.0 ± 2.6	15.4 ± 2.6	20.8 ± 8	10.7 ± 2.2	12.8 ± 2.9	3.5 ± 1.0	3.3 ± 0.9	2.8 ± 0.7	1.2 ± 0.4
Ped (mmHg)	7.8 ± 1.1	6.3 ± 0.8	8.1 ± 0.6	8.3 ± 1.4	6.9 ± 0.8	5.8 ± 0.8	6.4 ± 0.8	7.2 ± 2.0	6.8 ± 0.6	8.4 ± 2.4
Systolic parameters										
SW (mmHg*s)	1283 ± 169	1366 ± 108	1566 ± 235	1690 ± 104	1759 ± 265	1969 ± 202	1974 ± 272	2262 ± 147	2101 ± 309	2574 ± 198
dP/dt_max_ (mmHg/s)	6369 ± 542	6372 ± 642	7866 ± 762	7679 ± 577	9259 ± 1067	10,067 ± 1363	12,057 ± 1134	12,341 ± 652	14,107 ± 1026	15,189 ± 1227
PRSW (mmHg)	71.3 ± 2.5	81.8 ± 4.9	71.3 ± 5.0	80.1 ± 5.1	79.3 ± 5.9	94 ± 5.4	93.2 ± 4.5	*112.5 ± 4.7	97.7 ± 3.0	**133.8 ± 9.1
Diastolic parameters										
dP/dt_min_ (mmHg)	-6369 ± 542	-6513 ± 577	-7089 ± 762	-7338 ± 413	-7191 ± 713	-7517 ± 735	-6359 ± 756	-6218 ± 284	-7111 ± 627	-6020 ± 324
Tau (ms)	7.8 ± 0.3	7.4 ± 0.3	7.3 ± 0.4	6.9 ± 0.3	6.1 ± 0.3	5.6 ± 0.2	5.8 ± 0.4	5.1 ± 0.1	5.6 ± 0.3	5.4 ± 0.4

Significant differences between TRM4^+/+^and TRPM4^-/-^are indicated as bold values

HR, heart rate; SV, stroke volume; CO, cardiac output; Ved, end-diastolic volume; Ves, end-systolic volume; Ped, end-diastolic pressure; SW, stroke work; dP/dt_max_, peak rate of pressure rise; -dP/dt_min_, peak rate of pressure decline; PRSW, preload recruited stroke work; Tau, relaxation time constant (Weiss method). Values are expressed as means ± SEM

*p < 0.05, **p < 0.01

**Fig. 5:**
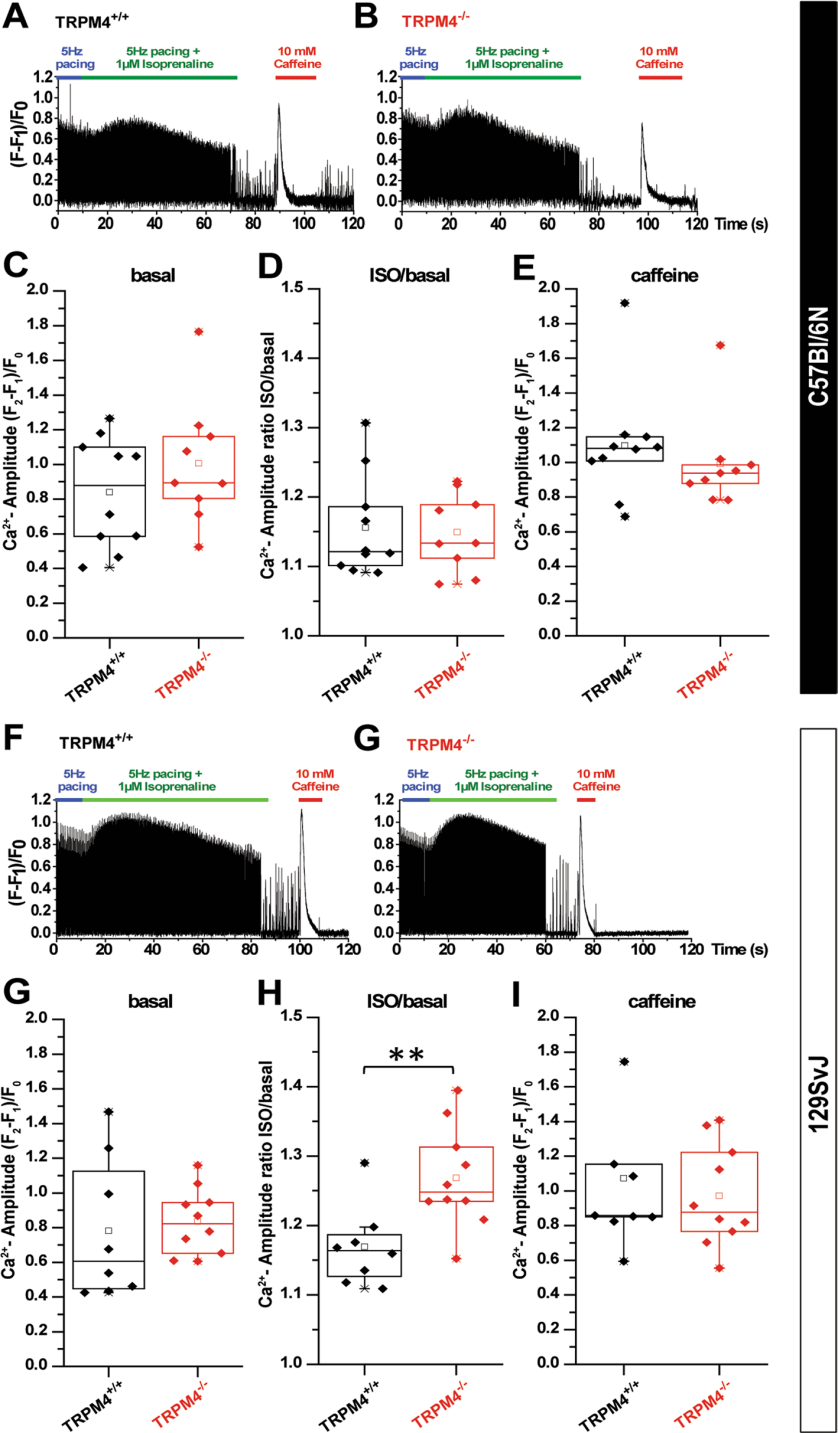
Analysis of Ca^2+^ transient in cardiomyocytes from Trpm4^+/+^ and Trpm4^−/−^ mice on the C57Bl/6N (**a**–**e**) or 129SvJ (**f**–**j**) background. (**a**–**b**) representative examples illustrating the time-course of experiments to test the effect of isoprenaline (1 µM) on electrically evoked Ca^2+^ transients (5 Hz). After the application of isoprenaline, the pacing was seized and cells were stimulated with caffeine (10 mM) to induce Ca^2+^ release. Analysis of the basal amplitude of electrically-evoked Ca^2+^ transients (**c**), the relative effect of isoprenaline (**d**) and the amplitude of the caffeine evoked transient (**e**) in cells from Trpm4^+/+^ and Trpm4^−/−^ cardiomyocytes from C57Bl/6 N mice. **f**–**j** show similar experiments but from cardiomyocytes from 129SvJ mice. Amplitudes of Ca^2+^ transients were calculated as follows: F2 represents the peak of the Ca^2+^ transient, F1 is the base of the Ca^2+^ transient, and F0 is the fluorescence value in a resting cell, before the cell is stimulated. Thus (F2-F1)/F0 gives the net amplitude of the Ca^2+^ transients normalized to the loading of the cell (F0). In all our traces background fluorescence (fluorescence value of a cell-free field) is subtracted from the data. Each data point represents averaged values from cells of biologically independent mice (10 WT and 9 KO animals on C57Bl/6N and 8 WT and 10 KO animals on 129SvJ background. Values are expressed as means ± SEM. **p < 0.01

To evaluate whether the differences in inotropic response in Trpm4^–/–(129SvJ)^ and Trpm4^/–(Bl/6N)^ mice after β-adrenergic receptor stimulation in comparison to their corresponding Trpm4^+/+^ controls are due to changes in Ca^2+^ signaling in cardiomyocytes isolated from the different mouse strains, we performed measurements of [Ca^2+^]_i_ in electrically paced cardiomyocytes from Trpm4^–/–(Bl/6N)^ and Trpm4^–/–(129SvJ)^ mice and WT littermates (Fig. 5a, b, f and g). The resting Ca^2+^ values (F1/F0) were identical between TRPM4 knockout and wildtype animals both in the 129SvJ and C57Bl/6N strain (resting Ca^2+^ values in C57Bl/6N strain: Trpm4^+/+(Bl/6N)^: 1.25 ± 0.11 (*n* = 10), Trpm4^–/–(Bl/6N)^ 1.33 ± 0.4 (*n* = 9), resting Ca^2+^ values in C57Bl/6N strain before iso stimulation: Trpm4^+/+(Bl/6N)^ 1.22 ± 0.13 (*n* = 10), Trpm4^–/–(Bl/6N)^ 1.33 ± 0.1 (*n* = 9), resting Ca^2+^ values in 129SvJ strain: Trpm4^+/+(129SvJ)^ 1.34 ± 0.1 (*n* = 8), Trpm4^–/–(129SvJ)^ 1.35 ± 0.17 (*n* = 10), resting Ca^2+^ values in 129SvJ strain before iso stimulation: Trpm4^+/+(129SvJ)^ 1.45 ± 0.1 (*n* = 8), Trpm4^–/–(129SvJ)^ 1.45 ± 0.17 (*n* = 10). During steady-state electrical pacing the amplitude of electrically evoked Ca^2+^ transients (systolic Ca^2+^ rise) was not different between Trpm4^–/–(Bl/6N)^ and Trpm4^+/+(Bl/6N)^ cardiomyocytes (Fig. 5c). Furthermore, after catecholamine stimulation with isoproterenol (1 µM) the amplitude of electrically evoked Ca^2+^ transients was significantly increased to the same extent in Trpm4^–/–(Bl/6N)^ and Trpm4^+/+(Bl6//N)^ cardiomyocytes (Fig. 5d). In Trpm4^–/–(129SvJ)^ cardiomyocytes the amplitude of electrically evoked Ca^2+^ transients were not different compared to Trpm4^+/+(129SvJ)^ cardiomyocytes under steady-state electrical pacing. However, the effect of catecholamine stimulation with isoproterenol (1 µM) (Fig. 5h and i) was enhanced in Trpm4^–/–(129SvJ)^ versus Trpm4^+/+(129SvJ)^ cardiomyocytes, which was in line with previously reported findings in Trpm4^–/–^ mice of the 129SvJ genetic background [21].

Since differences in Ca^2+^ signaling and contractility have been reported between C57Bl/6N and 129SvJ mouse strains before, which were attributed to differences in the expression of regulators of cardiomyocyte Ca^2+^ homeostasis such as voltage-gated L-type channels, the SERCA2a ATPase or others [41] we tested whether the expression of SERCA2a and of Ca_v_β_2_, the predominant accessory subunit of cardiac L-type channels [23, 42], might be differently expressed between cardiomyocytes from the two mouse strains. However, the densitometric analysis revealed no significant differences in these proteins between the two mouse strains (Fig. 1d, e). Notably, we observed significant differences in the abundance of TRPM4 proteins, which were 78 ± 10% higher in wild-type mice on the 129SvJ genetic background compared to the C57Bl/6N strain (Fig. 1c). Furthermore, we found no difference in the caffeine-evoked Ca^2+^ amplitude between cardiomyocytes of the 129SvJ strain and the C57Bl/6N strain indicating that the amount of Ca^2+^ which can be released from the SR is not different in cardiomyocytes of these two mouse strains (Fig. 5e, j).

## Discussion

Our previous studies have suggested that the activation of the cation channel TRPM4 in cardiomyocytes reduces the driving force for L-type mediated Ca^2+^ influx. The global deletion of Trpm4 in the mouse model resulted in increased cardiac contractility under β-adrenergic stimulation, both under healthy conditions [21, 37] and after induction of myocardial infarction [12]. Our current results suggest that in healthy mice this effect is critically dependent on the genetic background of the mouse model being studied. Indeed, both in an inducible cardiomyocyte-specific Trpm4^flox/flox^; αMHC-Cre^ERT2^ mouse model as well as in globally TRPM4-deficient mice on the same C57Bl/6N genetic background no increase in inotropy during β-adrenergic stimulation could be detected. Of note, a difference in the protocol used in this study is that the pressure–volume catheter is placed in the left ventricle via the apex, while in Mathar et al. the catheter was introduced into the left ventricle via the carotid artery with the chest closed [21]. The latter, previously published, PV loop measurements showed an increased ISO-evoked improvement of contractility in Trpm4^–/–^ mice on the 129SvJ background [21], which we could faithfully reproduce in this study, in global Trpm4^–/–(129SvJ)^ mice in comparison to Trpm4^+/+(129SvJ)^ littermates using open-chest insertion of the catheter via the apex. Determination of left ventricular function revealed a significantly increased β-adrenergic inotropy, as well as a significant increase in stroke volume during isoproterenol stimulation in Trpm4^–/–(129SvJ)^ mice in comparison to Trpm4^+/+(129SvJ^), controls. This indicates that the difference in the methodological approach cannot explain why an increase in ISO-evoked cardiac contractility was not observed upon TRPM4 deletion in healthy mice of the C57Bl/6N strain in our study. Since this phenomenon was confined to Trpm4^–/–(129SvJ)^ mice, we conclude that the phenotype of increased β-adrenergic inotropy depends on the genetic background, as well as on the health/disease status of the mice.

The influence of genetic background on cardiac functions has been demonstrated in several other studies. The genetic homogeneity of inbred strains provides ideal conditions for the investigation of causal physiological relationships. The most commonly used genetic background in the analysis of cardiac function is that of the C57Bl/6 mouse, the first mouse strain that was fully sequenced [24]. Most genetically modified mouse lines were generated from embryonic stem cells from inbred strains of the 129 background and then backcrossed to the genetic background of C57Bl/6 mouse strains. However, there are genetic divergences between different inbred strains, which are often paid little attention and can lead to confusing results when comparing different inbred strains [6]. For example, C57Bl/6 mouse strains have a single renin gene, Ren-1c, while 129SvJ mice have two renin genes, Ren-1d and Ren-2. This is associated with e.g. higher blood pressure in 129SvJ compared to C57Bl/6 mice [19]. Further studies reported differences in cardiac function between different inbred strains [2, 3, 27, 30, 31, 35, 38, 41]. Hemodynamic analysis of eight commonly used inbred mouse strains showed differences in cardiac contractility that were pronounced during acute hypoxia or β-adrenergic blockade [2]. The β-adrenergic blockade inhibited cardiac function more strongly in 129X1/SvJ than in the C57Bl/6J strain suggesting that 129X1/SvJ mice have a higher sympathetic basal tone than C57Bl/6J mice, which in addition would explain desensitization-induced lower cardiac β1 adrenoceptor density in 129X1/SvJ animals compared to C57Bl/6J mice [31]. Differences in contractility and relaxation between the 129SvJ and C57Bl/6 J strain with and without ISO treatment were reported using hemodynamic measurements [41]. Similar studies with comparative analysis of the cardiac contractile function of the C57Bl/6N strain have not been reported to our knowledge. The distinction between N and J strain is of importance for the analysis of cardiac function particularly in the disease state. A recent study could demonstrate in detail that the defective mitochondrial transhydrogenase (NnT) in C57Bl/6J ameliorates pressure overload-induced cardiac failure because of differences in antioxidative capacity [25].

Interestingly, differences in the expression of proteins affecting the intracellular Ca^2+^ transient were also observed between different inbred strains. Quantitative PCR analysis showed a significantly higher expression of RyR2 and an enhanced expression of SERCA2a in 129/SvJ mice compared to C57Bl/6 [41]. This could potentially affect intracellular Ca^2+^ homeostasis, as Shah and colleagues had pointed out. They recognized that cardiomyocytes from 129/SvJ mice have a higher sarcoplasmic reticulum (SR) “Ca^2+^-load” and a lower SR “Ca^2+^-leak” compared to C57Bl/6 mice [31]. However, under a frequency-dependent stimulation, no increase in systolic Ca^2+^ amplitude could be detected between the cardiomyocytes of the two strains, as well as no difference in SR Ca^2+^ release [31]. Also, in our hands, no obvious differences in the Ca^2+^ handling between 129SvJ and C57Bl/6N cardiomyocytes could be observed. In contrast, we found that the expression of TRPM4 proteins is about 78 ± 10% higher in wild-type mice on the 129SvJ genetic background compared to the C57Bl/6N strain. This interesting finding could indicate that the TRPM4 protein is more physiologically relevant, particularly with respect to inotropic response, in the left ventricle of 129SvJ mice than in C57Bl/6N mice. Mathar et al. suggested that the inotropic phenotype of TRPM4^−/−^ mice depends essentially on a modification of Ca^2+^ dependent excitation–contraction coupling in TRPM4^−/−^ mice. They found that the ISO-induced increase of the evoked Ca^2+^ transient is significantly larger in TRPM4^−/−^ cardiomyocytes, compared to WT cardiomyocytes. Considering that differences in Ca^2+^ signaling in cardiomyocytes from different mouse strains have been shown before [31], we studied catecholamine evoked rise in systolic Ca^2+^ transients in cardiomyocytes of both genotypes and mouse strains. Whereas the ISO-evoked increase of the Ca^2+^ transient was significantly larger in TRPM4^−/−^ cardiomyocytes in the 129SvJ strain compared to TRPM4^+/+^ controls, which reinforces our previous analysis [21], we could not find a significant difference between the two genotypes in the C57Bl/6 N background. In our attempts to gain mechanistic insights into the differences in TRPM4-dependent Ca^2+^ signaling between the two strains, we analysed protein expression of the L-Type Ca^2+^ channel subunit Ca_V_β_2_ and SERCA2a proteins, whose expression level and activity regulate diastolic SR Ca^2+^ load [7]. However, our findings show neither divergences in Ca_V_β_2_ and SERCA2a protein expression nor differences in caffeine-induced SR Ca^2+^ depletion in cardiomyocytes from 129SvJ compared to C57Bl/6N mice indicating that SR Ca^2+^ load is not different in ventricular cardiomyocytes between the strains. Taken together, these results confirm our previous analysis. The finding that cardiac TRPM4 expression in C57Bl/6N was drastically (∼ 80%) lower compared 129SvJ mice suggests that TRPM4 is a relatively less important determinant of the driving force of L-type Ca^2+^ entry in C57Bl/6N cardiomyocytes, as compared to 129SvJ cardiomyocytes.

However, we cannot exclude that also other factors that determine ISO-evoked Ca^2+^ rise and cardiac contractility on the organismal level might be different between TRPM4-deficient mice on the respective genetic background. An example of such discrepancies was reported in the study of Vignier et al. who investigated the LMNA p.H222P mutation leading to Emery-Dreifuss muscular dystrophy (EDMD) in humans in C57BL/6JRj and 129S2/SvPas background. This mutation is associated with arrhythmia-related symptoms resulting in cardiac dilation and heart failure. In contrast to C57^Lmna p.H222P^ mice, 129^Lmna p.H222P^ mice showed exacerbated arrhythmia susceptibility making 129^Lmna p.H222P^ mice a more relevant model for evaluating therapeutic strategies for EDMD [40]. The studies above as well as our findings emphasize that the analysis of null alleles or disease-related mutations in multiple mouse strains is recommendable. Ultimately, the most adequate species and strain need to be chosen to study distinct physiological or pathophysiological processes [2, 13, 38, 41] and to test potential therapeutic strategies. Obviously, the 129SvJ strain is more suitable to study TRPM4-dependent processes in the heart compared to the C57Bl/6N strain where the homeostatic expression level is comparably low.

Overall, it can be concluded that a genotype–phenotype context of an isolated genetic background is not easily generalized for different inbred mouse strains [28, 33]. F1 hybrids could be a useful alternative to investigate the influence of TRPM4 deficiency in a more complex genetic background. In a previous study by Uhl et al., TRPM4-deficient 129B6F1 hybrids were used, which under β-adrenergic stimulation with isoproterenol achieved an increased positive inotropic response in isolated left ventricular papillary muscles compared to wild-type 129B6F1 hybrids [37]. However, it remains to be investigated whether cardiac contractility in the TRPM4-deficient 129B6F1 hybrid mouse also leads to an increased β-adrenergic positive inotropy in vivo. The extent of relevance of TRPM4 deletion may also be differently pronounced between healthy mice and different degrees of disease status. Whereas in this study the inotropic response was unaltered in Trpm4^–/–(Bl/6N)^ mice of the C57Bl/6N background, it could be shown that the β-adrenergic reserve was preserved in Trpm4^–/–(Bl/6N)^ mice after permanent LAD ligation and significantly higher compared to Trpm4^+/+(Bl/6N)^ leading to an increased survival rate [12]. A potential beneficial effect of inactivating or deleting TRPM4 in other cardiac disease models in the mouse or other species has not been investigated so far.

From a translational point of view, a single isogenic mouse strain is limited in its genetic diversity and may not reflect the physiological responses of the human polymorphic population [14]. The analysis of a null mutation in multiple mouse strains offers the opportunity to increase the genetic diversity of the mouse model, which allows comparison with the broad spectrum of human genetic heterogeneity [28]. Thus, the concept of inhibiting TRPM4 channels or suppressing their expression needs to be explored in different genetically diverse populations of patients suffering from cardiac failure.
